# Spurious-Free Shear Horizontal Wave Resonators Based on 36Y-Cut LiNbO_3_ Thin Film

**DOI:** 10.3390/mi15040477

**Published:** 2024-03-30

**Authors:** Yushuai Liu, Kangfu Liu, Jiawei Li, Yang Li, Tao Wu

**Affiliations:** 1 School of Information Science and Technology, ShanghaiTech University, Shanghai 201210, China; liuysh2@shanghaitech.edu.cn (Y.L.);; 2 Shanghai Institute of Microsystem and Information Technology, Chinese Academy of Sciences, Shanghai 200050, China; 3 University of Chinese Academy of Sciences, Beijing 100864, China

**Keywords:** lithium niobate (LiNbO_3_), resonator, high electromechanical coupling coefficient, shear horizontal wave

## Abstract

This article presents lithium niobate (LiNbO_3_) based on shear horizontal (SH0) resonators, utilizing a suspended structure, for radio frequency (RF) applications. It demonstrates the design, analysis, and fabrication of SH0 resonators based on a 36Y-cut LiNbO_3_ thin film. The spurious-free SH0 resonator achieves an electromechanical coupling coefficient ($k_{t}^{2}$) of 42.67% and a quality factor (Q_r_) of 254 at the wave-propagating orientation of 0° in the 36Y-cut plane.

## 1. Introduction

For the next generation of mobile handsets, cognitive radios, and Internet of things, radio frequency (RF) front ends need high functionality and flexibility simultaneously, within the limited RF spectrum [1,2]. The implementation of piezoelectric resonators, particularly surface acoustic wave (SAW) and bulk acoustic wave (BAW) resonators, favor a technology framework that can provide high performance for different applications [3,4]. The use of DC-DC converters with a piezoelectric resonator as the only energy-storage element has demonstrated the need for a high electromechanical coupling coefficient $k_{t}^{2}$ and for spurious-free modes. Spurious-free modes can improve the operating range of DC–DC converters [5]. The $k_{t}^{2}$ is proportional to the voltage-conversion efficiency [6]. The spurious modes near the pass-band remain a major challenge as they lower the $k_{t}^{2}$ of the intended resonance and create in-band ripples and out-of-band spurious responses in filter applications [7].

Many piezoelectric devices have been investigated, such as surface acoustic wave (SAW) devices, thin-film bulk acoustic resonators (FBARs), and laterally vibrating resonators (LVRs). In recent decades, these resonators, which are based on different kinds of piezoelectric material, including aluminum nitride (AlN) [8,9], lead zirconate titanate (PZT) [10,11], doped AlN [12,13,14,15], and lithium niobate (LiNbO_3_) [16,17,18], have attracted wide research interest. Among these platforms, AlN FBARs have demonstrated 7% $k_{t}^{2}$ [19], but it is challenging to implement multiple wide resonant frequencies on the same chip with FBARs because of the thickness extensional mode. Furthermore, SAW devices cannot be integrated into CMOS processes and have limited scalability for higher frequencies over 3 GHz due to their low acoustic velocity [1]. The low piezoelectric constant of AlN limits the maximum $k_{t}^{2}$ to approximately 6% [20]. Recently, Sc-doped aluminum nitride (AlScN) was studied to improve the piezoelectric constant of AlN. A 24% Sc-doped two-dimensional resonant-rod resonator achieved a $k_{t}^{2}$ of 23.9%, but it had a low quality factor, of 101 [21]. A relatively high Sc concentration of up to 43% can help enhance the $k_{t}^{2}$, but the structure of AlScN loses all its piezoelectric properties close to 60% Sc [22]. Additionally, high Sc concentrations can also cause high density in anomalously oriented grains, which causes $k_{t}^{2}$ and Q degradation [23,24].

Unlike FBARs and SAW devices, LVRs can cover multiple frequencies on the same wafer, and are also compatible with the CMOS process. The LVRs leveraging transferred LiNbO_3_ thin films have been developed to feature higher $k_{t}^{2}$ and Q at the same time. The LiNbO_3_ LVRs based on various acoustic modes, including symmetric (S0), shear horizontal (SH0), and first-order antisymmetric (A1) modes, have exhibited extraordinarily high $k_{t}^{2}$ (>20%) and Q of up to several thousand at RF [7,24,25,26,27,28,29,30]. Despite their impressive performance, these devices have not fully harnessed their pronounced piezoelectric properties due to the spurious response in LiNbO_3_ resonators. The spurious response originates from various kinds of unwanted mode. It will be challenging to fully utilize the piezoelectric properties of LiNbO_3_ to achieve resonators with large $k_{t}^{2}$ and Q. In particular, the in-band ripples caused by the spurious mode adjacent to the intended mode make it difficult to obtain the maximum bandwidth and minimum insertion loss simultaneously. Therefore, the suppression of these spurious modes is of great significance for the application of LiNbO_3_ LVRs. Recently, a few studies focused on the origin and suppression of spurious modes in LiNbO_3_ LVRs. Suppression techniques for spurious modes have been developed using modified edge shapes [31], length-controlled electrode configurations [1] and 2-electrode-array designs [32] in SH0 LiNbO_3_ LVRs, and weighted electrode configurations in S0 LiNbO_3_ LVRs [33], as well as the method based on the recessed electrodes in LiNbO_3_ A1 resonators [34].

Specifically, this paper investigates the shear horizontal modes of 0-order (SH0) in thin plates of 36Y-cut LiNbO_3_ to determine the trade-offs between different resonator-structure parameters in order to suppress the spurious response and improve the $k_{t}^{2}$. The 36Y-cut was selected because it has a major advantage in terms of the piezoelectric stress coefficient e_16_ compared with other orientations of LiNbO_3_ [1,24,35,36,37]. It can help excite the SH0 mode with its large electromechanical coupling coefficient. Additionally, most studies on SH0 resonators have focused on the X-cut because it is more readily available from wafer vendors, and it can also couple with other vibration modes easily [1,31,36,38,39]. A few studies on SH0 resonators based on 36Y-cut lithium niobate on an insulator (LNOI) focused on temperature-stability analysis [18,40]. Among the different modes of Lamb wave resonator, the 0-th-order shear horizontal (SH0) mode possesses the highest $k_{t}^{2}$. However, few examples exist in the literature that make full use of the advantages of 36Y-cut LiNbO_3_ to achieve a $k_{t}^{2}$ of more than 40% and spurious-free modes simultaneously. In this work, we explore the impact of various geometrical parameters, such as the pitch, length, and width of the IDT electrodes on the $k_{t}^{2}$ of a SH0-mode resonator in 36Y-cut LiNbO_3_ and demonstrate passband spurious-free devices, with a highest achieved $k_{t}^{2}$ of 42.6%. In addition, the influence of the electrode parameters on the suppression of the spurious modes is also discussed. Finally, spurious-free LVRs with high $k_{t}^{2}$, which we fabricated in this study, are characterized.

## 2. Design and Analysis

### 2.1. Excitement of SH0 Mode in LiNbO_3_

In this work, SH0 mode is focus because of the largest intrinsic electromechanical coupling factor ($K_{ij}^{2}$) in LiNbO_3_ for this particular mode and low velocity dispersion over a wide range of film thicknesses [41]. The 36Y-cut LiNbO_3_ has a large piezoelectric-stress-constant component of −4.48 (C/m^2^) in e_16_, which can excite shear horizontal mode effectively [42]. The complete rotated e-matrix for 36Y-cut LiNbO_3_ is as follows [17]:

(1) $$e=\left[\begin{array}{ccc} 0 & 0 & 0\\ -1.65 & -2.30 & 2.57\\ -1.94 & -1.59 & 4.53 \end{array}\;\;\;\;\begin{array}{ccc} 0 & 0.12 & -4.48\\ 0.47 & 0 & 0\\ -0.26 & 0 & 0 \end{array}\right]\;C/m^{2}$$

**Figure 1:**
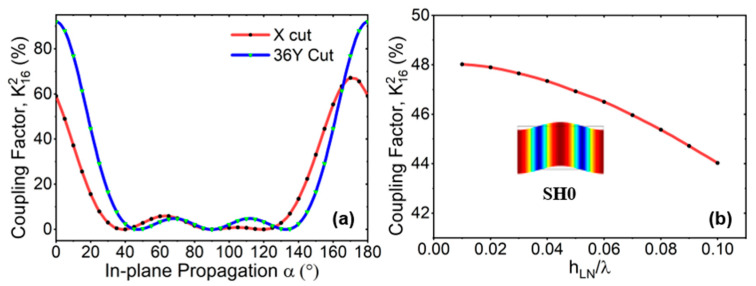
The $K_{16}^{2}$ of (**a**) numerical simulation varies with in-plane-propagation direction *α* in X-cut and 36Y-cut and (**b**) FEA simulation of SH0 mode with different normalized thickness of LiNbO_3_ (h_LN_) and wavelength (λ) under open and short conditions when *α* = 0° for SH0 mode.

To quantitatively compare different orientations, the electromechanical coupling $K_{ij}^{2}$ is studied under a quasi-static approximation, where *i* is the electric field direction and *j* is the stress component. Figure 1a shows $K_{16}^{2}$ versus in-plane-propagation direction *α* for the SH0 mode based on X and 36Y cuts. Compared with commonly used X-cut [1,24,35,36,37], 36Y-cut has larger $K_{16}^{2}$. Here, the Euler rotated angle is (*α*, 54, 0) for 36Y-cut. The electrode-arrangement direction is along the *x*-axis direction after Euler rotation, and α represents the in-plane direction of wave propagation. The $K_{ij}^{2}$ is defined as follows [43]:

(2) $$K_{ij}^{2}={e_{ij}}^{2}/\left({\varepsilon_{ii}}^{T}\times {s_{jj}}^{E}\right)$$

where *e* is the piezoelectric coefficient, $\varepsilon^{T}$ is the permittivity under constant stress, and $s^{E}$ is elastic compliance under constant electric field. Obviously, when α is around 0, the $K_{16}^{2}$ of the SH0 mode is extremely high. Therefore, SH0 mode can be excited efficiently in this case. Based on these results (i.e., α=0°), Figure 1b presents the $K_{16}^{2}$ of SH0 with the different normalized LiNbO_3_ thickness (h_LN_/λ) within 0.1 (wavelength λ equals twice pitch of IDT). The $K_{16}^{2}$ of SH0 mode gradually decreases as the h_LN_/λ increases. Here, $K_{16}^{2}$ ($K_{16}^{2}=\left({v_{p}}^{2}-{v_{s}}^{2}\right)/{v_{s}}^{2}$) is calculated using the velocities of the same acoustic mode under the open ($v_{p}$) and short ($v_{s}$) conditions. The vibration-mode shape of SH0 is also shown in Figure 1b.

### 2.2. Suppression of High-Order SH0 Spurious Mode

**Figure 2:**
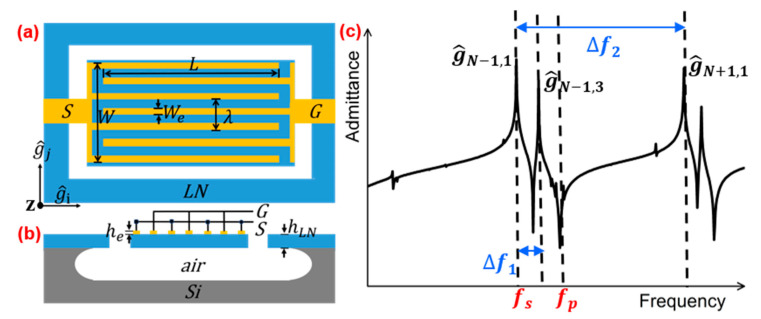
(**a**) Top view and (**b**) cross-section view of conventional electrode configuration. (**c**) Admittance response of a spurious-mode resonator with N top electrodes. The $∆f_{1}$ represents the frequency gap between the fundamental mode ${\hat{g}}_{N-1,1}$ and high-order longitudinal modes ${\hat{g}}_{N-1,3}$, and $∆f_{2}$ represents the frequency gap between the fundamental mode ${\hat{g}}_{N-1,1}$ and high-order transverse modes ${\hat{g}}_{N+1,1}$.

The SH0 wave on the bulk material leaks into the substrate, which can be mitigated by utilizing a suspended thin-film structure [44]. Several studies of the suppression of spurious modes focused on piezoelectric resonator [45], where longitudinal and transverse indicate the direction along and perpendicular to the propagation direction. The top view and cross-section view of conventional electrode configuration for SH0 resonator are shown in Figure 2a,b. Here, W and L are the width and length of the suspended plate, respectively. The W_e_, W_p_, and λ represent the width of the electrode, pitch, and the wavelength, respectively. Neglecting the in-plane an-isotropic, the resonant frequencies of all the acoustic modes in a plate can be expressed by:

(3) $$f_{i,j}=\frac{v_{0}^{'}\left|{\hat{g}}_{\left(i,j\right)}\right|}{2\pi }=v_{0}^{'}\sqrt{{\left(\frac{i}{2W}\right)}^{2}+{\left(\frac{j}{2L}\right)}^{2}}$$

(4) $${\hat{g}}_{i,j}={\hat{g}}_{i}+{\hat{g}}_{j}$$

where *i* and *j* are the wave vectors of the longitudinal and transverse modes and $v_{0}^{'}$ is the phase velocity of the acoustic wave. For a device with N electrodes, ${\hat{g}}_{N-1,1}$ is the desired main mode. In operation, electric fields introduced by the top electrode induce periodic strain and stress fields, forming acoustic modes of various orders, as depicted in Figure 2c [24]. To form spurious-free filters, the nature of spurious modes in a typical LiNbO_3_ LVR needed to be investigated first, before spurious-mode-mitigation feature could be developed.

**Figure 3:**
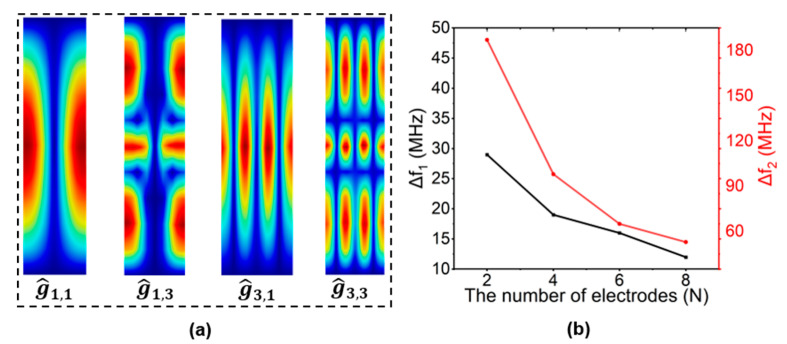
(**a**) Displacement-mode shapes with ${\hat{g}}_{1,1}$, ${\hat{g}}_{1,3}$, ${\hat{g}}_{3,1}$, and ${\hat{g}}_{3,3}$ and (**b**) spectral spacing $∆f_{1}$ and $∆f_{2}$ with the different numbers of interdigitated electrodes (N), while h_LN_ = 0.75 µm, L = 150 µm, W_p_ = 10 µm, h_e_ = 0.2 µm, and W_e_/W_p_ = 50%.

To visualize the displacement of shear horizontal modes of various orders, COMSOL finite element analysis (FEA) was used to simulate the eigenmodes in 3D LiNbO_3_ modes (Figure 3a). Various SH0 shape modes of ${\hat{g}}_{1,1}$, ${\hat{g}}_{1,3}$, ${\hat{g}}_{3,1}$ and ${\hat{g}}_{3,3}$ are shown, with a mode order denoting the number of half-wavelength periodicities in the longitudinal and transverse directions.

#### 2.2.1. The Number of Electrodes (N)

For a resonator with a particular number of electrodes (more than 2), spurious modes occur at various frequencies. When the number of electrodes increases, the higher-order transverse (${\hat{g}}_{\left(N+1\right),1},{\hat{g}}_{\left(N+3\right),1}\ldots$) and longitudinal (${\hat{g}}_{\left(N-1\right),3},{\hat{g}}_{\left(N-1\right),5}\ldots$) modes are often positioned closer to the desired mode (${\hat{g}}_{\left(N-1\right),1}$). The minimum number of interdigitated electrodes (N = 2) would make the value of $∆f_{1}$ and $∆f_{2}$ reach maximum ($∆f_{1}$ and $∆f_{2}$ represent the frequency gap between the fundamental mode ${\hat{g}}_{N-1,1}$ and high-order longitudinal mode ${\hat{g}}_{N-1,3}$, the fundamental mode ${\hat{g}}_{N-1,1}$, and high-order transverse mode ${\hat{g}}_{N+1,1}$, respectively), as shown in Figure 3b. This can contribute to distancing and attenuating higher transverse and longitudinal modes, and it can also create a large spurious-free range for comprising filters. Consequently, the main mode distances from and attenuates higher-order longitudinal and transverse modes to the greatest extent when the number of electrodes N = 2, creating the largest spurious-free space.

#### 2.2.2. The Pitch of Electrodes (W_p_)

**Figure 4:**
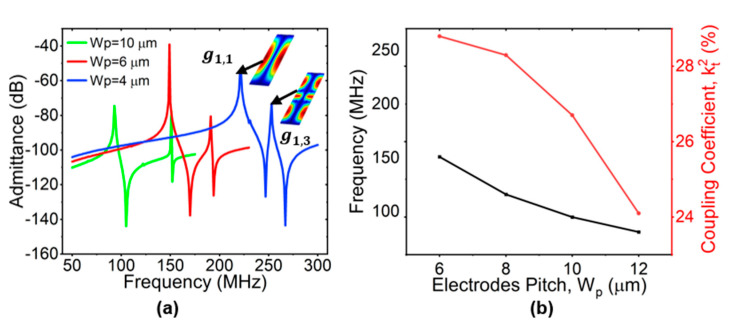
(**a**) Simulated admittance with different W_p_ settings of 4 µm, 6 µm, and 10 µm, respectively, and (**b**) simulated frequency of ${\hat{g}}_{1,1}$ and coupling coefficient $k_{t}^{2}$ with the changes in electrode pitch $W_{p}$, while N = 2, h_LN_ = 0.75 µm, L = 100 µm, h_e_ = 0.2 µm, and W_e_/W_p_ = 50%.

The simulated admittance curves with different pitches are shown in Figure 4a, where electrodes are N = 2, h_LN_ = 0.75 µm, L = 100 µm, h_e_ = 0.2 µm, and W_e_/W_p_ = 50%. The ${\hat{g}}_{1,1}$ and ${\hat{g}}_{1,3}$ are labeled on the curve when W_p_ = 10 µm, as an example. As expected, as the pitch increased, ${\hat{g}}_{1,3}$ is moved far away from the desired ${\hat{g}}_{1,1}$. Considering the fabrication accuracy and the suppression of the parasitic mode, W_p_ = 10 µm was selected for the subsequent analysis. The simulated variations of frequency and $k_{t}^{2}$ with W_p_ are shown in Figure 4b. They both increased significantly when W_p_ decreased. Larger W_p_ values led to smaller frequency and $k_{t}^{2}$, but spurious-free modes. In the early stage, the $k_{t}^{2}$ was derived from the thickness mode, and the value was close to the definition of $K_{ij}^{2}$ [46]. Next, the expression of $k_{t}^{2}$ was improved by fitting the measured value according to the Butterworth Van Dyke (BVD) model, which was applicable to laterally vibrating piezoelectric resonators [47]. The $k_{t}^{2}$ is defined using the series ($f_{s}$) and parallel ($f_{p}$) resonant frequency [48]:

(5) $$k_{t}^{2}=\frac{\pi^{2}}{8}\frac{f_{p}^{2}-f_{s}^{2}}{f_{s}^{2}}$$

#### 2.2.3. The Lengths of Electrodes (L)

**Figure 5:**
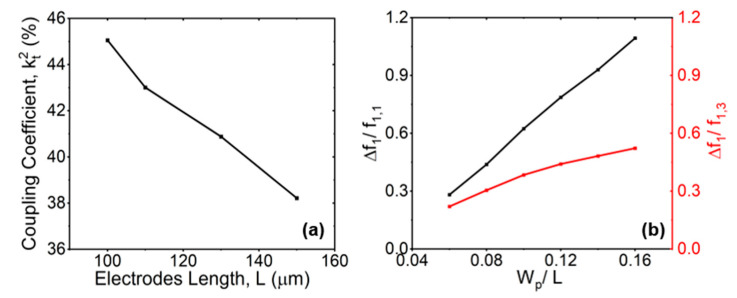
Simulated (**a**) coupling coefficient $k_{t}^{2}$ of $g_{1,1}$ with the changes in electrode length L, while N = 2, h_LN_ = 0.75 µm, W_p_ = 10 µm, h_e_ = 0.2 µm, and W_e_/W_p_ = 30%; (**b**) $∆f_{1}/f_{1,1}$ and $∆f_{1}/\;f_{1,3}$ with the changes in W_p_/L.

The 3D COMSOL FEA was used to analyze the suppression of transverse modes based on different electrodes’ lengths. We set W_e_/W_p_ = 50%, W_p_ = 10 μm. The values of the simulated $k_{t}^{2}$ of the ${\hat{g}}_{1,1}$ mode at different electrode lengths L are shown in Figure 5a. The $k_{t}^{2}$ was negatively correlated with the electrode length, indicating that longer L caused lower $k_{t}^{2}$. This can be explained by the fact that higher-order acoustic waves can be scattered from the resonant cavity in the transverse direction, thereby eliminating the spurious mode and improving $k_{t}^{2}$ of fundamental mode when L decreases [31]. Figure 5b presents a no-dimensional analysis of the ratio of $∆f_{1}/f_{1,1}$ and $∆f_{1}/f_{1,3}$ with different W_p_/L. The $∆f_{1}$ was the same at fixed W_p_ and L, but $f_{1,1}$ and $f_{1,3}$ were different. As L gradually increased or W_p_ gradually decreased, the curves of $∆f_{1}/f_{1,1}$ and $∆f_{1}/f_{1,3}$ gradually overlapped. This indicates that the $f_{1,1}$ and $f_{1,3}$ were becoming closer, which also meant that the influence of the spurious mode on the main mode increased. The ratio of $∆f_{1}$ and $f_{1,1}$ or $f_{1,3}$ was related to $W_{p}/L$, which can be explained by Equation (3). In conclusion, larger L not only caused lower $k_{t}^{2}$ in ${\hat{g}}_{1,1}$, but it also led to a tighter frequency gap between ${\hat{g}}_{1,3}$ mode and desired mode ${\hat{g}}_{1,1}$, which probably led to spuriousness in passband. Larger L also had more spurious modes and lower $k_{t}^{2}$.

In general, a resonator with a minimum number of interdigitated electrodes (N = 2) would attenuate higher-order spurious modes and create a larger spurious-free tuning range for wideband oscillators and RF filters. However, a single two-electrode resonator would have a very small static capacitance (C_0_) in comparison to the feedthrough or parasitic capacitance (C_f_) between probing pads [49]. The measured results of single resonators typically produce high rates of uncertainty, particularly when C_0_ is smaller than C_f_. To attain a higher static capacitance (C_0_) for better impedance matching, an array of parallel-connected two-electrode resonators can be employed [48,50].

## 3. Fabrication and Measurement Results

### 3.1. Fabrication Process

**Figure 6:**
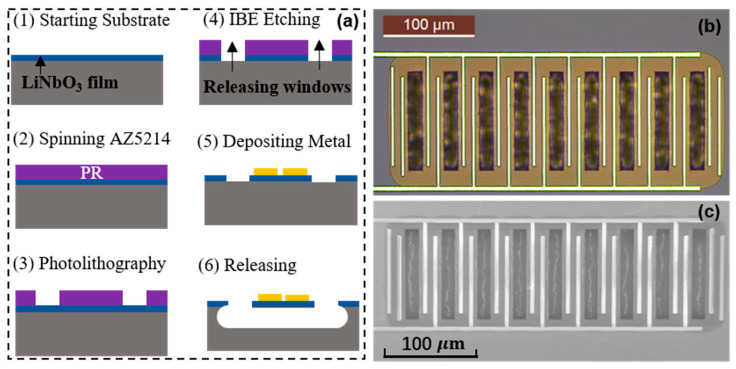
(**a**) The fabrication process for LiNbO_3_ lateral vibrating resonators: (1) start with 36Y-cut LiNbO_3_ material, (2) deposit PR as the etching mask, (3) conduct the first lithography to define the releasing windows, (4) perform LiNbO_3_ etching with IBE, (5) conduct the second lithography to define the Al electrodes, deposit 10 nm Ti and 200 nm Al, lift off, and (6) release the resonator with XeF_2_. (**b**) Optical image and (**c**) SEM image of a fabricated LiNbO_3_ resonator device (M = 8).

Figure 6a shows the fabrication process of the LiNbO_3_-film resonator for SH0 modes. Firstly, a 36Y-cut LiNbO_3_ film 0.75 μm in thickness was transferred onto a high-resistivity Si wafer. The film was procured from Fluoroware (now part of Entegris). Before the ion-beam etch (IBE) process, hard baking (115 °C for 10 min) was performed on the AZ5214 to harden the photoresist (PR) to serve as the mask for the etching of the LiNbO_3_.

A bias voltage of 300 V was used in the IBE-etching process, and the etching rate was approximately 13 nm/min [51]. In addition, the temperature variation in the whole process was minimized to avoid thermal stress. Next, the photoresist mask (AZ5214) was removed with Piranha, and 10 nm Ti and 200 nm Al were subsequently defined on top of the LiNbO_3_ thin film as the IDT electrodes, using a lift-off process. To suspend the resonator structure, the Si under the LiNbO_3_ devices was removed with XeF_2_-based isotropic dry etching.

One of the fabricated LiNbO_3_ SH0 devices is shown in Figure 6b,c. The L of the fabricated devices was 100 µm. Multiple groups with identical two-electrode resonators were connected in parallel to increase the C_0_, which tuned the impedance matching with the RF terminal. For the fabricated resonator, the dummy electrodes were implemented on the edges of the resonators to ensure that the structure was symmetrical and that identical resonances were obtained for all the parallel resonators [32].

### 3.2. Measured Results and Discussion

#### 3.2.1. Measurement Analysis of N and L

**Figure 7:**
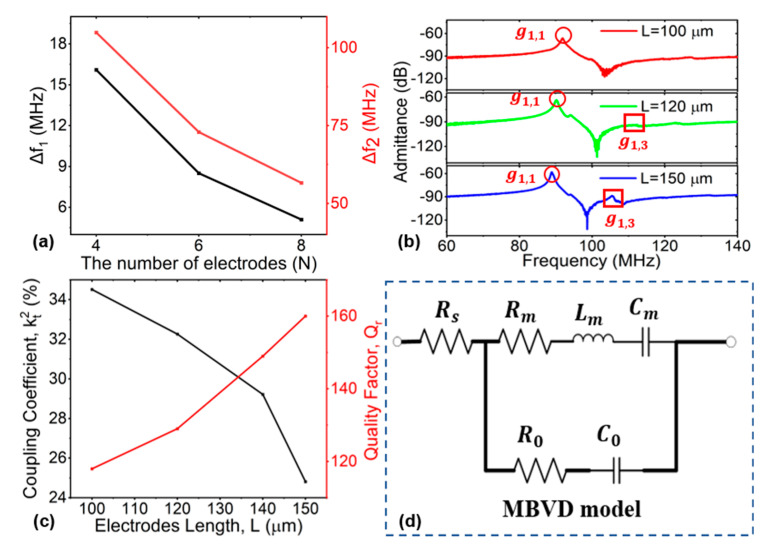
(**a**) Measured frequency gaps of $∆f_{1}$ and $∆f_{2}$ with differences in electrode number N. (**b**) Measured de-embedded admittance responses with electrode length L set as 100, 120, and 150 µm, respectively. (**c**) Measured coupling coefficient Q_r_ and $k_{t}^{2}$ with the changes in electrode length L, while M = 6, N = 2, h_LN_ = 0.75 µm, W_p_ = 10 µm, h_e_ = 0.2 µm, and W_e_/W_p_ = 30%. (**d**) MBVD model.

The S-parameter data of the one-port LiNbO_3_ LVRs were measured by a network analyzer (Keysight N5234B). The feedthrough capacitances of the signal-grounding probing pads and routing connection were responsible for lowering the experimentally observed $k_{t}^{2}$. Thus, the extraction of accurate $k_{t}^{2}$ from the results measured from a single resonator requires the de-embedding of the feedthrough or parasitic capacitance [49]. The S-parameter matrix was converted to a Y-parameter matrix to extract the admittance of the device under test (DUT), and the net admittance of the resonator was then obtained by de-embedding the open structure on the same chip from the DUT [52]. The measured frequency gaps of the $∆f_{1}$ and $∆f_{2}$ with different electrode numbers Ns are shown in Figure 7a. The lower N contributed to larger spurious-free frequency gaps, which was consistent with the simulated results shown in Figure 3b. Lower N values also caused lower excitement efficiency in the ${\hat{g}}_{1,3}$; therefore, the ${\hat{g}}_{1,3}$ mode was not present in the measured admittance at N = 2. The measured admittance responses with L = 100 µm, 120 µm, and 150 µm are shown in Figure 7b. With the electrodes’ lengths L increasing, ${\hat{g}}_{1,3}$ approached the desired ${\hat{g}}_{1,1}$, and the excitement efficiency of the spurious mode ${\hat{g}}_{1,3}$ also increased. The measured $k_{t}^{2}$ and Q_r_ with different L values are shown in Figure 7c,d. Larger L values also increased the quality factor Q_r_, which can be explained by the fact that the vibrational energy was better confined within the resonator body, and little escaped through the anchors [30]. However, the coupling coefficient $k_{t}^{2}$ decreased with increases in electrode length L.

The $k_{t}^{2}$ of the resonator can be calculated by identifying *f_r_* and *f_p_* using Equation (5), in line with common practice. The $k_{t}^{2}$ can be alternatively extracted by fitting the measured admittance with the MBVD model (Figure 7d) [53]. The model consists of the static capacitor C_0_, the motional resistor R_m_, the motional inductor L_m_, the motional capacitor C_m_, and the series resistance (R_s_). The R_s_ shows the resistance of the pads and electrodes, which is measured from test structures with shorted fingers [54]. The R_m_ represents the actual energy dissipation in a resonator. The L_m_ and C_m_ represent the interchangeable mechanical energy storage in a resonator, which can be expressed by referring to [8]. The quality factors (Q_r_) can be expressed as follows [3,24,55,56]:

(6) $$Q_{r}=\frac{f_{r}}{∆f_{3dB}}$$

The single-resonance MBVD fitting method is reliable for extracting circuit parameters in cases of spurious-free near-the-main-mode or low-coupling resonators, in which only the resonance (*f_s_*) and antiresonance (*f_p_*) frequency peaks are fitted [3]. In this case, Q_r_ can be accurately obtained using the ratio of the frequency to the −3 dB frequency widths of the impedance response at *f_r_*, as in Equation (6).

#### 3.2.2. Measurement Analysis of W_p_

**Figure 8:**
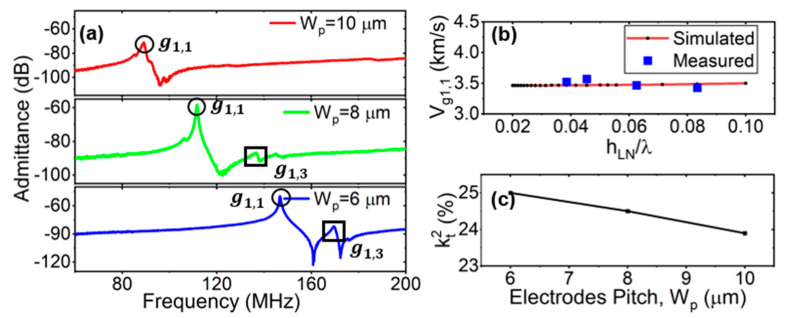
(**a**) Measured de-embedded admittance response with pitch W_p_ set as 4 µm, 6 µm, and 10 µm, respectively, while W_e_/W_p_ = 50%. (**b**) Simulated and measured phase velocity of ${\hat{g}}_{1,1}$ (V_g1,1_) with different h_LN_/λ. (**c**) Measured $k_{t}^{2}$ with different W_p_.

The device’s frequency responses as a function of pitches W_p_ are shown in Figure 8a. The main mode ${\hat{g}}_{1,1}$ and the spurious mode ${\hat{g}}_{1,3}$ near the main mode are labeled on the curves when W_p_ = 6 µm, 8 µm, and 10 µm, respectively. Similar to the simulated results shown in Figure 4, the interval between ${\hat{g}}_{1,3}$ and ${\hat{g}}_{1,1}$ increased when the W_p_ increased. Figure 8b shows the comparison with the simulated and measured phase velocity of the LVRs based on the 36Y-cut LiNbO_3_. The measured data were extracted through RF measurement. The phase velocity of the LiNbO_3_ operating in the ${\hat{g}}_{1,1}$ SH0 mode was about 3500 m/s. The operating frequency of the resonators was changed by varying the designed devices’ wavelengths. Although increases in the W_p_ suppressed the spurious modes of the devices, this eventually led to decreases in $k_{t}^{2}$, as shown in Figure 8c.

#### 3.2.3. Measurement Analysis of Electrode Coverage (W_e_/W_p_)

**Figure 9:**
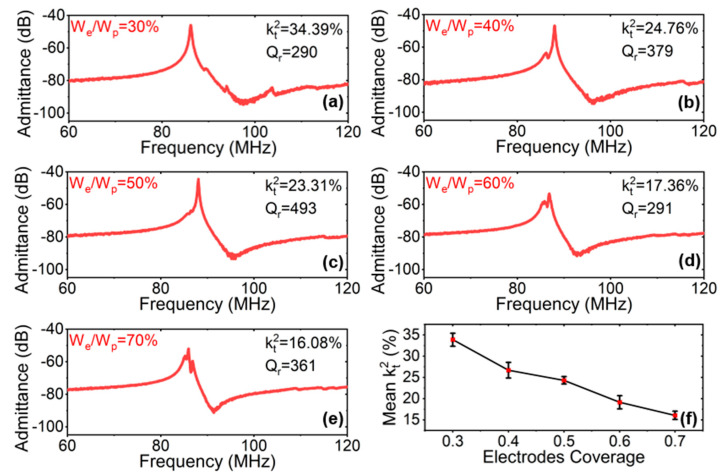
(**a**–**e**) Measured de-embedded admittance responses under different coverages (W_e_/W_p_), while M = 5, N = 2, h_LN_ = 0.75 µm, W_p_ = 10 µm, and h_e_ = 0.2 µm, and (**f**) measured mean coupling coefficient $k_{t}^{2}$ with the changes in electrode coverage.

Coverage can directly affect the capacitance per unit area under a given wavelength. Increases in this parameter facilitate the fabrication of more compact devices and reduce the need for arraying large numbers of resonators [57,58]. The $k_{t}^{2}$ depends on the electrode coverage (W_e_/W_p_) of the device, as it directly influences C_0_ and C_m_. Figure 9 shows the measured admittance response and MBVD model fitting with different coverages (W_e_/W_p_). The corresponding $k_{t}^{2}$ and resonant-quality factor Q_r_ are marked. The increasing of W_e_/W_p_ represents a reduction in the spacing between the electrodes, which caused the C_0_ to grow non-linearly as $C_{0}\propto 1/\left(1-W_{e}/W_{p}\right)$. At the same time, due to the increase in electrode area, the C_m_ increased linearly with the W_e_/W_p_ [54]. The $k_{t}^{2}$ dropped gradually when the coverage increased. The device with W_e_/W_p_ = 30% had the highest $k_{t}^{2}$. This was consistent with the analysis of electrode coverage in previous S0 resonators [58].

Five electrodes’ coverage values were investigated, and the respective Q_r_ values were recorded (Figure 9a–e). The relationship between device W_e_/W_p_ and Q_r_ is still under investigation [57]. Figure 9f illustrates the comparison between the values of the measured mean electromechanical coupling $k_{t}^{2}$ under different degrees of electrode coverage W_e_/W_p_. All had similar trends, in that smaller electrode coverage led to larger $k_{t}^{2}\;$values. The mean $k_{t}^{2}$ values varied from 33.9% to 16.1%, with the W_e_/W_p_ increasing from 0.3 to 0.7.

**Figure 10:**
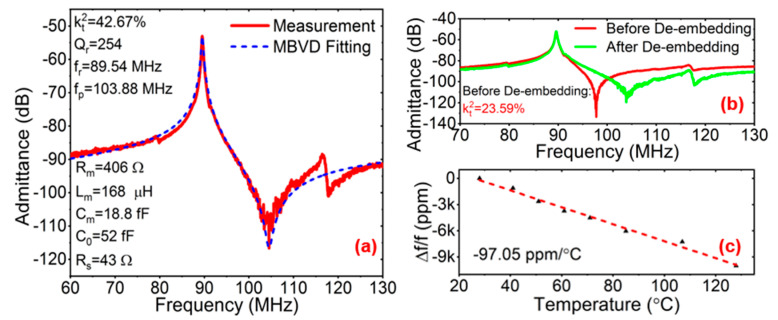
(**a**) Measured admittance response and MBVD fitting after de-embedding, (**b**) measured admittance response before and after de-embedding the effects of feedthrough capacitances and (**c**) temperature coefficient of frequency (TCF) for the device with W_e_/W_p_ = 0.3, W_p_ = 10 μm, M = 8, and L = 100 μm.

In this study, we finally explored high-$k_{t}^{2}$ and spurious-less LVRs based on a 36Y-cut LiNbO_3_/Si substrate, as shown in Figure 10a,b. It is worth mentioning that the equivalent electrical MBVD model is a behavioral model, which is only valid around the resonance frequency of a modeled resonator [59]. This means that it may have infinite configurations for the same response when not considering the physical properties of the individual resonator [60]. In order to ensure that the values of the MBVD fitting were within a reasonable range, we used a Keysight Technologies B1500A semiconductor analyzer device to measure the I–V curves of the pad and the routing connection. The contact losses were used to model the series resistor R_s_ (~43 Ω). Using the FEM simulation and the analysis results above, the cut angle of the LiNbO_3_ was optimized as 36°, and the in-plane propagation direction *α* was 0°. The device was designed with an electrode coverage of W_e_/W_p_ = 0.3, the electrode array M = 8, and electrode length L = 100 μm. The fabricated LVRs were confirmed as having a $k_{t}^{2}$ of 42.67% after de-embedding. The temperature coefficient of frequency (TCF) was extracted by monitoring the shift in the series-resonance frequency as a function of temperature. Temperature measurements in the range of 28 °C to 128 °C were performed. Figure 10c shows the measured TCF for the fabricated SH0 resonator device. The extracted TCF was −97.05 ppm/°C, which is larger than that of pure AlN. This is attributable to the increased thermal expansion coefficients. Further temperature-compensation techniques can be implemented to improve the device TCF. The appearance of the spurious mode between the *f_s_* and the *f_p_* is attributable to a slight variation in the mechanical boundary conditions and, thus, resonant-frequency mismatch between individual resonators in the array [55]. The spurious mode can be eliminated by improving the fabrication accuracy to ensure that each resonance unit in the array has the same response.

Finally, Table 1 provides a comparison between our work and previous thin-film LiNbO_3_ LVRs. The A1 resonator has a higher frequency than the SH0 with the same fabrication accuracy because the A1 mode has a greater velocity than the SH0 mode. Due to the high e_16_, the X-cut and 36Y-cut can both achieve high $k_{t}^{2}$. Although the resonators in [54] exhibited the best $k_{t}^{2}$, they also have multiple spurious modes in the passband. As a result, the proposed 36Y-cut LiNbO_3_ SH0 resonators not only feature a simple process but show a well-balanced performance in terms of $k_{t}^{2}$ and spurious-mode suppression. Their operating frequency can be improved by fabricating electrodes with shorter wavelengths using E-beam lithography for higher-frequency applications. The fabrication process is described in [54].

## 4. Conclusions

In this work, we designed and analyzed the performance of a 36Y-cut LiNbO_3_ thin film based on resonator devices. By configuring the length and width of the IDT electrode, the transverse spurious mode ${\hat{g}}_{1,3}$ was suppressed efficiently. In addition, the influence of the electrode coverage on the coupling coefficient $k_{t}^{2}$ of the SH0 mode was discussed. The method of suppressing the transverse spurious mode and the influence of the coverage on the coupling were verified by the experimental device’s fabrication and characterization. The fabricated devices achieved a peak electromechanical coupling of 42.67% and a quality factor (Q_r_) of 254. Future research could focus on improving the Q value of the array. Potential methods for improving the Q value of the array include the improvement of the etching sidewall and roughness, vacuum encapsulation, and addressing imperfections and non-uniformities among the elements in the array.

## Figures and Tables

**Table 1:** Comparison of previous works.

Designs	Cut	Mode	$k_{t}^{2}(\%)$	Q *	*f_r_* (MHz)	Spurious Modes
[1]	X-cut	SH0	20.6	1064	~150	No
[55]	X-cut	SH0	17.1	915	85.41	No
[61]	X-cut	SH0	32	798	907.87	Yes
[54]	X-cut	SH0	41	1900	288	Yes
[34]	128Y-cut	A1	28	692	~2.8 GHz	No
[30]	X-cut	S0	30.7	5110	50.9	Yes
This Work	36Y-cut	SH0	42.67	254	89.54	No

* note: different papers may have different definitions of $k_{t}^{2}$ and Q.

## Data Availability

Not applicable.
